# N6‐methyladenine‐related genes affect biological behavior and the prognosis of glioma

**DOI:** 10.1002/cam4.3574

**Published:** 2020-12-02

**Authors:** Shanqiang Qu, Zhixin Chen, Bin Liu, Jin Liu, Huafu Wang

**Affiliations:** ^1^ Department of Neurosurgery Nanfang Hospital, Southern Medical University Guangzhou China; ^2^ Department of Neurosurgery The First Affiliated Hospital of Sun Yat‐sen University Guangzhou China; ^3^ Department of Emergency Surgery The First Affiliated Hospital of Nanchang University Nanchang China; ^4^ Department of Orthopedic Lishui People's Hospital (The Sixth Affiliated Hospital of Wenzhou Medical University Lishui China; ^5^ Department of Neurosurgery Lishui People's Hospital (The Sixth Affiliated Hospital of Wenzhou Medical University Lishui China; ^6^ Department of Clinical Pharmacy Lishui People's Hospital (The Sixth Affiliated Hospital of Wenzhou Medical University Lishui China

**Keywords:** neoplasms, nomograms, prognosis

## Abstract

**Background:**

Although aberrant expression of N6‐methyladenine (m^6^A) methylation‐related genes contribute to tumorigenesis in many solid tumors, the prognostic value of the m^6^A‐related genes and their correlation with clinicopathological features in gliomas need advanced study.

**Methods:**

The clinical and sequencing data of 288 patients with glioma were extracted from Chinese Glioma Genome Atlas database. By univariate and multivariable Cox regression analysis, the m^6^A‐related prognostic genes were identified, and their correlation with clinicopathological features was further analysis. A nomogram was constructed by R software and the performance of it was assessed by calibration and time‐dependent receiver operating characteristic curve.

**Results:**

Nine m^6^A‐related genes were identified as independent prognostic factors, which were mostly enriched in RNA splicing, regulation of immune response and vesicle‐mediated transport. By expression value and regression coefficient of these genes, we constructed risk score of each patient, which was highly associated with clinicopathological features. Kaplan–Meier curve showed that the prognosis of patients with high‐risk scores was significantly worse than that with low‐risk scores (HR = 4.30, 95% CI = 3.16–5.85, *p* < 0.0001). A nomogram was constructed based on the nine m^6^A‐related genes signature and clinicopathological features with well‐fitted calibration curves (c‐index = 0.82), showing high specificity and sensitivity (area under the curve for 1‐, 3‐, and 5‐years survival probability = 0.874, 0.918, and 0.934).

**Conclusions:**

A nine m^6^A‐related genes signature was identified in gliomas. The m^6^A‐related risk score is a novel prognostic factor for patients with glioma, and is associated with clinicopathological features. Moreover, the nomogram based on the nine m^6^A‐related genes signature and clinicopathological features had good efficacy in predicting the survival probability.

## Graphical abstract

The nine m6A‐related genes risk score was identified as a new potential prognostic biomarker in gliomas. The nomogram based on the nine m6A‐related genes signature and clinicopathological features had good efficacy in predicting the survival probability, which is pivotal to design individual therapy for patients with glioma

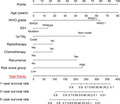

## 1 INTRODUCTION

Glioma, a brain tumor that originates from the neuroepithelium, is a frequent primary intracranial malignant tumor. According to the 2016 World Health Organization (WHO) classification system, gliomas can be divided into two categories: low‐grade glioma (LGG, WHOI‐II) and high‐grade glioma (HGG, WHOIII‐IV).^1^ HGG, which are characterized by high invasiveness and heterogeneity, accounts for more than half of intracranial primary malignant tumors. The statistical report of the Central Brain Tumor Registry of the United States showed that the patients with glioma between 2011 and 2015 in the United States accounted for 26% of all intracranial tumor patients and 81% of all intracranial malignant tumor patients.^2^ Currently, the effective treatments for gliomas include surgery, radiotherapy, and chemotherapy. However, these combination therapy has a limited effect on patients with glioma and the overall survival (OS) of patients is still unsatisfactory. The median survival is only 12–15 months for glioblastomas and 2–5 years for anaplastic gliomas.^3^ Therefore, it is critical to explore the molecular mechanisms underlying glioma and find effective prognostic biomarkers.

N6‐methyladenine (m^6^A) methylation modification of mRNA in eukaryotes was first reported as early as 1974.^4^ However, it is not until recently that its regulatory mechanism is gradually revealed. Increasing studies have confirmed that m^6^A modifications are played an important role in regulating tumor initiation and progression.^5^, ^6^, ^7^ As we all known, m^6^A methylation is the most common modification in the internal sequence of eukaryotic RNA, which depends on the precise regulation of three kinds of molecules, such as writer like METTL14, eraser like ALKBH5 and reader like YTHDF3.^8^ However, it is a very complex biological process that involves various molecular abnormalities. In recent years, it has been found that m^6^A‐demethylase was upregulated and involved in malignant biological process in gliomas.^9^, ^10^, ^11^ For instance, Sicong Zhang et al reported that high expression of RNA demethylase ALKBH5 promotes glioma stem cells (GSC) self‐renewal and proliferation.^10^ Additionally, Qi Cui et al observed that knockout of fat mass and obesity‐associated protein (FTO) inhibits GSC self‐renewal and proliferation and promotes GSC differentiation.^12^ Abhirami Visvanathan confirmed that METTL3 overexpression are essential for GSC maintenance by regulating A‐to‐I and C‐to‐U editing.^13^ However, the correlation between expression levels of m^6^A methylation‐related genes and clinicopathological features of gliomas have not been comprehensively investigated, and their prognostic value for gliomas is still further worthy to explore.

Therefore, we extracted the clinical and RNA sequencing data of patients from Chinese Glioma Genome Atlas (CGGA) database, which included more than 2000 glioma samples from Chinese patients, to systematically analyze the correlation between expression of m^6^A methylation‐related genes and clinicopathological features, and their prognostic value for patients in this study. Furthermore, a prediction model would be constructed based on the hub m^6^A‐related genes and clinical data of patients, which is of great significant to design individual treatment strategy for patients with different prognosis.

## 2 METHOD

### 2.1 Data collection

We extracted the clinicopathological data and RNA sequencing data of patients with glioma from the data set 3^14^ ^,^ ^15^ of CGGA (http://cgga.org.cn/). Finally, we included 288 patients with glioma into this study.

### 2.2 Select m^6^A methylation‐related genes and data processing

First of all, we searched the candidate genes related to the word of “m^6^A methylation” from the GeneCards database (https://www.genecards.org/), and then, screened the m^6^A‐related genes with the relevant score >1. Finally, we obtained 67 m^6^A‐related genes. The sequencing data of m^6^A‐related genes were normalized by “preprocessCore” package.

### 2.3 Identification of the m^6^A methylation‐related genes of patients' prognosis

The m^6^A methylation‐related genes affecting patients' prognosis was identified by univariate Cox analysis. The gene with *p* < 0.05 was further included into multivariate Cox regression analysis for identifying the independent prognostic genes of patients.

### 2.4 The correlation between risk score and clinicopathological features of patients

The risk score was calculated by the expression levels of m^6^A methylation‐related gene and its coefficient. The formula of risk score is as follows:

$$\text{Risk}\;\text{score}=\sum_{i=1}^{n}\left({\text{Gene}}_{i}*{\text{Coef}}_{i}\right).$$

Chi‐square test was performed to explore the correlation between clinicopathological features and risk score. The risk score on prognosis of patients with glioma was analyzed by Kaplan–Meier survival analysis.

### 2.5 Construction and validation of nomogram

Based on clinicopathological features and risk score, a composite model is constructed by using “Survival” package and “rms” package. Calibration curves and time‐dependent receiver operating characteristic (ROC) curve are performed to test the accuracy of the prognostic model.

### 2.6 Biological function analysis

To explore the biological function of m^6^A methylation‐related genes in gliomas, we retrieved the 25 most relevant co‐expressed genes in the TCGA‐CNS/Brain (https://www.cboportal.org/).^16^ ^,^ ^17^ The m^6^A methylation‐related genes of patients' prognosis and the co‐expressed genes were further entered into Gene Ontology (GO) and Kyoto Encyclopedia of Genes and Genomes (KEGG) pathway enrichment analysis. Additionally, we used the String database to observe the protein–protein interaction between proteins encoded by m^6^A methylation‐related genes (https://string‐db.org/).^18^

### 2.7 Statistical analysis

All data were analyzed by SPSS software (version 23.0, IBM Corp.) and R software version 3.6.1. Chi‐square test was used for classified variables. The mRNA levels of m^6^A‐related genes in gliomas with different WHO grades were compared by One‐way ANOVA, and the pairwise comparison between groups was performed by least significant difference test. An independent sample *t* test and the Mann–Whitney *U* test were used to compare the expression levels in gliomas for age, gender, isocitrate dehydrogenase (IDH) status, and 1p/19q status. A calibration plot was drawn by comparing nomogram‐predicted survival probability with the Kaplan–Meier‐estimated survival probability. Moreover, we performed time‐dependent ROC curve analysis using “survival ROC” package. Survival curves were depicted using the Kaplan–Meier method and compared using the log–rank test. All tests were two‐sided, and *p* < 0.05 were considered to be statistically significant (*p < 0.05; **p < 0.01; ***p < 0.001; ****p < 0.0001).

## 3 RESULTS

### 3.1 Characteristic of patients with glioma

In this study, we identified 288 patients with RNA‐sequencing data and complete clinicopathological data from CGGA database. The detailed characteristics of all patients were showed in Table S1. The average age and median survival of patients were 43.23 ± 0.72 years and 25.63 months, respectively. The 1‐, 3‐, and 5‐year survival probability for patients were 62.44%, 42.71%, and 32.99%, respectively.

### 3.2 Identification of m^6^A methylation‐related genes of patients' prognosis

To identify the m^6^A methylation‐related genes affecting patients' prognosis, 67 candidate genes were obtained from GeneCards database. Due to the exclusion of LOC100418723 and RIDA, which were lacked of the sequencing data, the remaining 65 genes were finally included into univariate analysis for patients' prognosis. A total of 40 genes remained (*p* < 0.01). Consequently, the 40 significant genes (*p* < 0.01) were further entered into multivariate analysis.

Finally, the multivariate analysis identified nine independent prognostic genes (Table S2). The biological function and hazard regression coefficients of the nine m^6^A‐related genes signature were shown in Table S3. Next, risk score of each patient was calculated by expression value and the coefficients of each independent prognostic gene. The calculation formula is as follows: Risk score = ADCY3*(−1.51) + ALKBH5*(3.34) + DGCR8*(1.61) + FHL2*(−2.21) + FTO*(2.12) + PICALM*(2.04) + TRMT112*(5.16) + YTHDF2*(1.76) + YTHDF3*(2.70).

### 3.3 The expression level of nine m^6^A methylation‐related genes in gliomas

**FIGURE 1:**
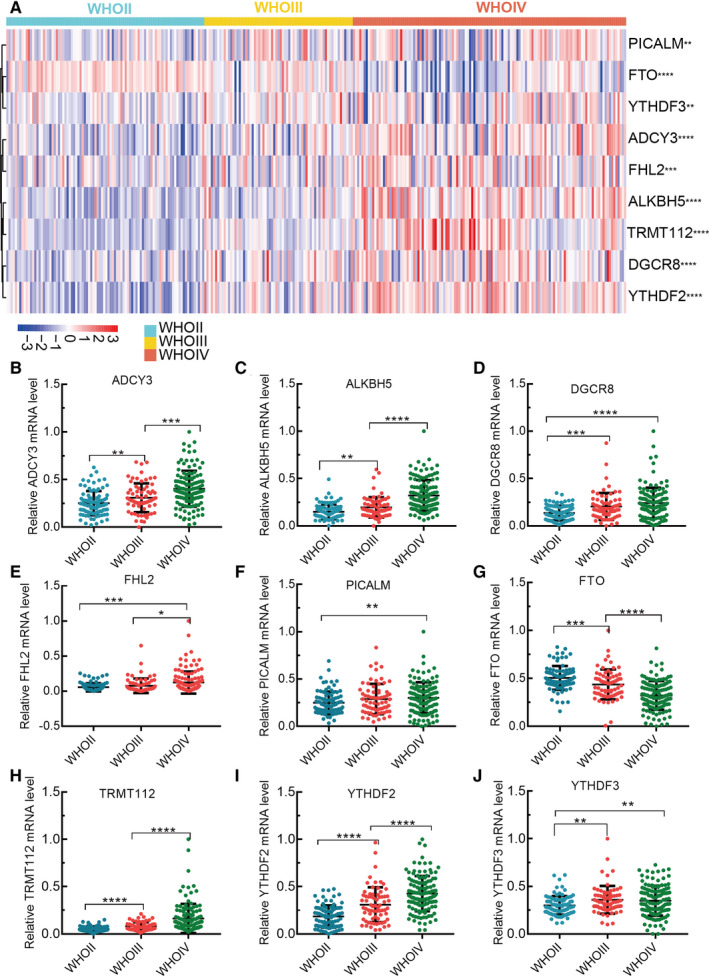
Differential expression of m^6^A‐related genes in different grade gliomas. A, The differential expression of m^6^A‐related genes was present as heatmap; (B‐J) The statistical plots of the m^6^A ‐related genes expression in different grades gliomas

We statistically analyzed the sequencing data of nine m^6^A methylation‐related genes in 288 patients with glioma. The correlation between the expression level of each gene and WHO grade was presented as heatmaps (Figure 1A). Meanwhile, we also compared the differential expression of nine m^6^A methylation‐related genes in different WHO grades. The results showed that each m^6^A methylation‐related gene was significantly associated with WHO grades (Figure 1B–J).

Moreover, we also used the glioma data of TCGA to analyze the differential expression of each gene between WHO grade II and WHO grade III glioma. Except for the genes of FHL2 and YTHDF3, there were significant differential expression of the other genes between WHO grade II and WHO grade III glioma (Figure S1A–I).

To identify protein expression level of each gene in normal brain tissue and glioma, immunohistochemistry (IHC) were retrieved from the Human Protein Atlas database, which revealed the expression level of m^6^A methylation‐related proteins (No data found for ADCY3, DGCR8 and YTHDF3) (Figure S2A–F).

To detect whether nine m^6^A methylation‐related genes were associated with IDH status and 1p/19q status, all patients were stratified by glioma grades (LGG and HGG). The relationship between IDH status and expression levels of each m^6^A methylation‐related gene in LGG and HGG were presented as heatmaps (Figure S3A,B). As shown in Figure S3A, the differential expression of FTO, ALKBH5, DGCR8, ADCY3, and FHL2 was related to IDH status in LGG. Likewise, as shown in Figure S3B, the differential expression of FTO, ALKBH5, DGCR8, ADCY3, PICALM, TRMT112, and FHL2 was related to IDH status in HGG. The relationship between 1p/19q status and expression levels of each m^6^A methylation‐related gene in LGG and HGG were presented as heatmaps (Figure S3C,D). As shown in Figure S3C, the differential expression of ALKBH5, YTHDF2, TRMT112, PICALM, and FHL2 was related to 1p/19q status in LGG. As shown in Figure S3D, the differential expression of FTO, ALKBH5, YTHDF2, PICALM, TRMT112, and FHL2 was related to 1p/19q status in HGG.

### 3.4 The correlation between risk score and clinicopathological features in gliomas

To evaluate the correlation between risk score and clinicopathological features, all patients were divided into high‐ (*n* = 144) and low‐risk group (*n* = 144) according to the median risk score, and the correlation between risk score and each clinicopathological feature was analyzed by chi‐square test. The results indicated that risk score was significantly correlated with age (*p* = 0.041), WHO grade (*p* < 0.001), IDH status (*p* < 0.001), 1p/19q status (*p* < 0.001), radiotherapy (*p* = 0.022), and recurrence (*p* < 0.001) (Table 1).

**TABLE 1:** The correlation between the nine‐m^6^A‐related genes risk scores and clinicopathological parameters

Parameters	Cases (%)	m^6^A risk scores		*P*‐value
		Low risk	High risk	
Age(y)				
≥40	173 (60.1)	78	95	0.041
＜40	115 (39.9)	66	49	
Gender				
Male	180 (62.5)	85	95	0.224
Female	108 (37.5)	59	49	
WHO grade				
WHO II	92 (31.9)	80	12	<0.001
WHO III	69 (24.0)	42	27	
WHO IV	127 (44.1)	22	105	
IDH				
Mutation	154 (53.5)	99	55	<0.001
Wild‐type	134 (46.5)	45	89	
1p/19q				
Codel	60 (20.8)	54	6	<0.001
Non‐codel	228 (79.2)	90	138	
Radiotherapy				
Yes	244 (84.7)	129	115	0.022
No	44 (15.3)	15	29	
Chemotherapy				
Yes	171 (59.4)	75	96	0.012
No	117 (40.6)	69	48	
Recurrence				
Yes	208 (72.2)	26	54	<0.001
No	80 (27.8)	118	90	

**FIGURE 2:**
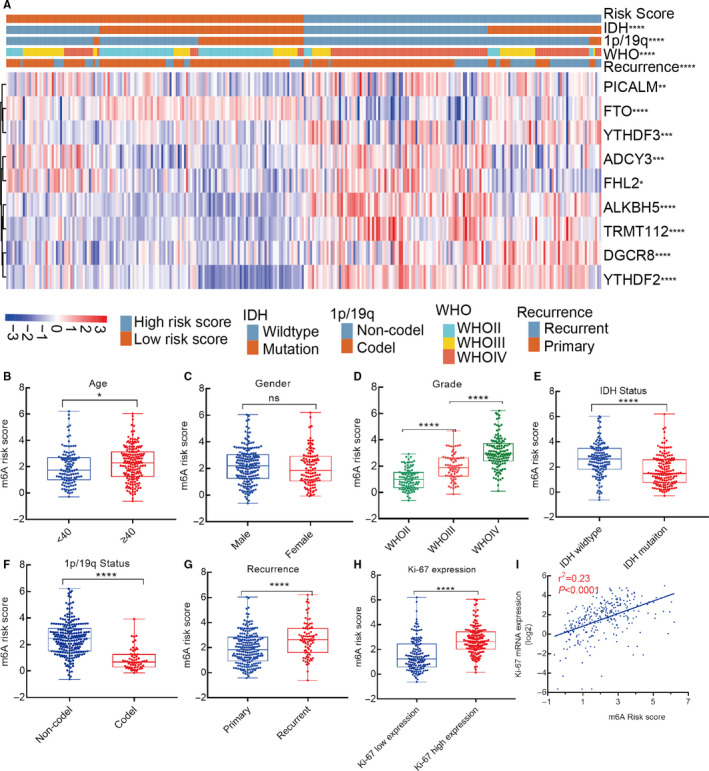
The correlation between risk score and clinicopathological features in glioma. A, Differential clinicopathological features in high‐ and low‐risk score; (B‐H) distribution of risk score in patients with glioma stratified by age, gender, grade, IDH status, 1p/19q status, recurrence, and ki‐67 expression; (I) scatter plot of m^6^A‐related risk score and its corresponding ki‐67 expression based on linear regression analysis (*r* ^2^ = 0.23, *p* < 0.0001)

The heatmap visually showed the correlation between risk score and clinicopathological features and genes expression in 288 patients with glioma (Figure 2A). We also further assessed the distribution of risk score in 288 patients stratified by age, gender, grade, IDH status, 1p/19q status, recurrence, and ki‐67 expression. Consistently, the results revealed that risk score was significantly associated with age, grade, IDH status, 1p/19q status, recurrence, ki‐67 expression, but not gender (Figure 2B–H). In addition, to clarify whether m^6^A‐related genes are related to malignant biological behavior, we draw a scatter plot to observe the correlation between risk score and ki‐67 mRNA levels, showing that m6A‐related genes are significant associated with tumor malignancy (*r* ^2^ = 0.23, *p* < 0.0001) (Figure 2I).

### 3.5 Biological function of nine m^6^A methylation‐related genes in gliomas

**FIGURE 3:**
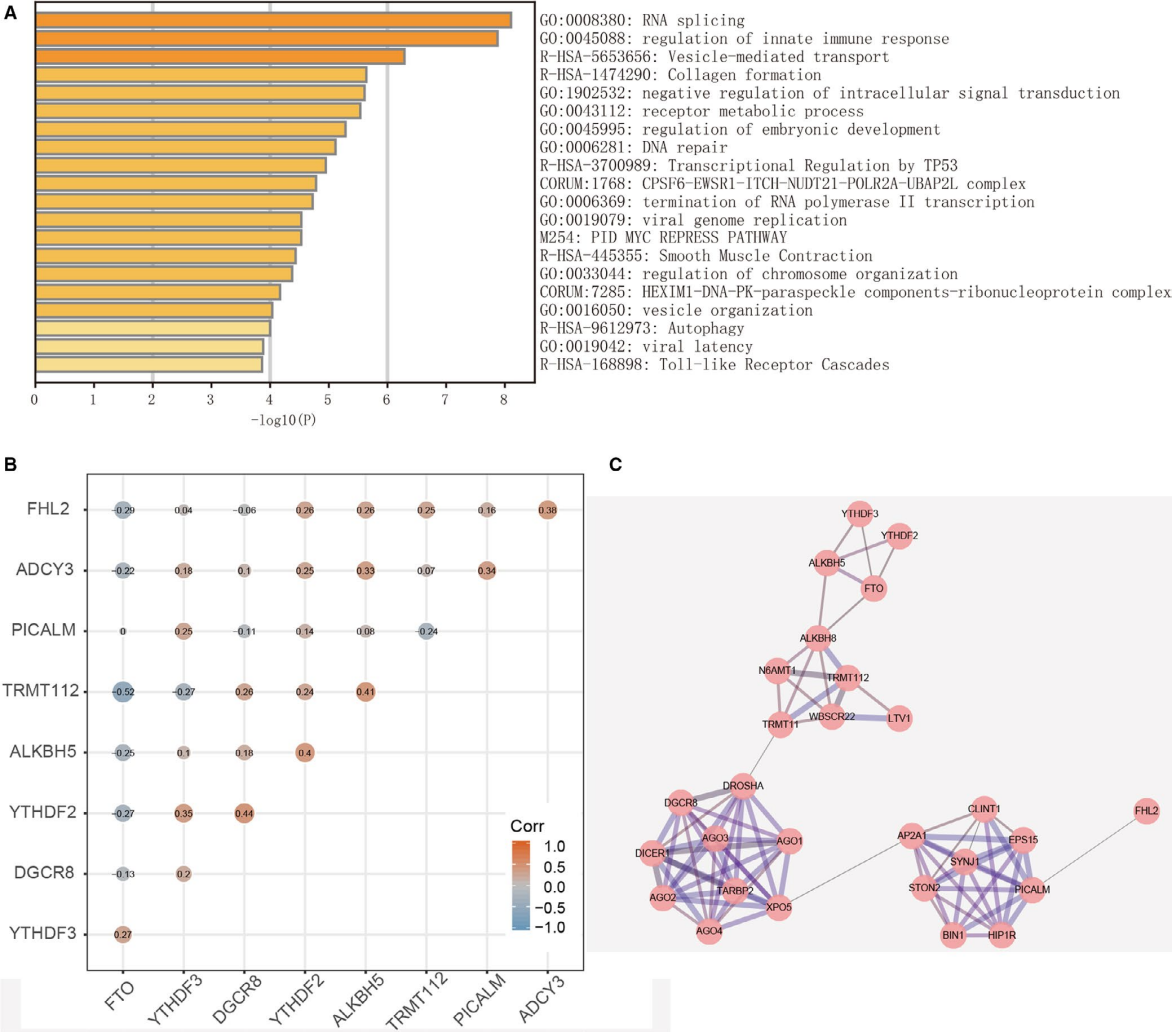
Interaction among m^6^A‐related genes and the involved functional processed in gliomas. A, Identification of functional processes involved in these genes by Gene Ontology (GO) and Kyoto Encyclopedia of Genes and Genomes enrichment analysis; (B) Spearman correlation analysis of the nine m^6^A‐related genes; (C) The visual protein–protein interaction network of m^6^A‐related protein by String database.

To further explore the biological processes of these genes in gliomas, we first obtained the most relevant 25 co‐expressed genes for each m^6^A methylation‐related gene in the TCGA‐CNS/Brain (http://www/cbioportal.org/). Then, we use GO and KEGG enrichment analysis to explore biological function of these genes, and found that these genes were mostly enriched in RNA splicing, regulation of immune response, vesicle‐mediated transport (Figure 3A).

In addition, we further calculated the coefficient between these genes by Spearman correlation test. The result was presented as heatmap (Figure 3B). To better understand protein–protein interactions among the nine m^6^A methylation‐related genes, Figure 3C clearly showed their interactive relationship through the String database.

### 3.6 Correlation between risk score and OS of patients with glioma

**FIGURE 4:**
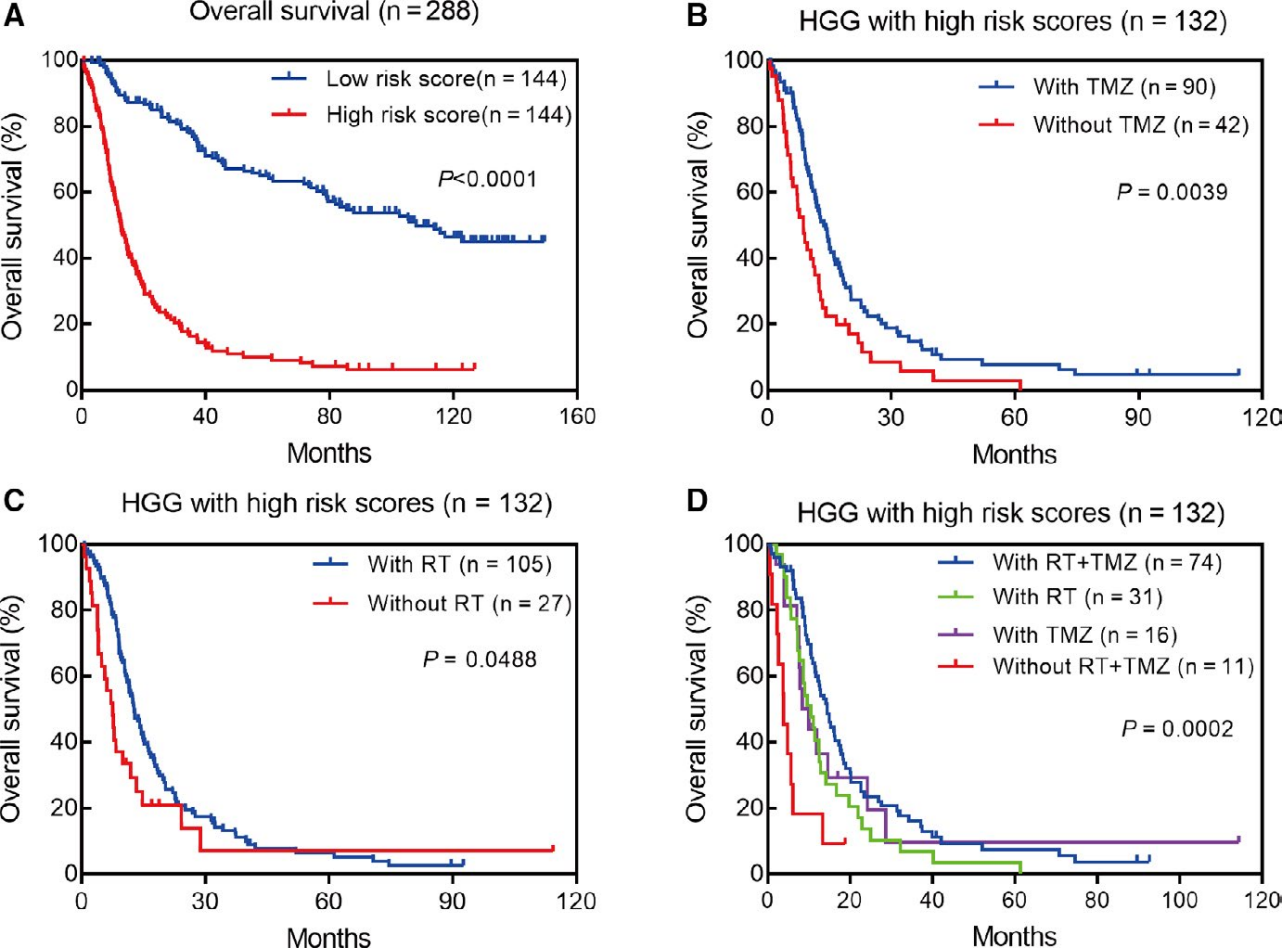
Prognostic value of m^6^A‐related genes in gliomas. A, Kaplan–Meier survival analysis of m^6^A‐related genes for overall survival; (B‐D) Patients with high‐risk scores could benefit from temozolomide chemotherapy and radiotherapy in high‐grade glioma (HGG)

To investigate the prognostic value of risk score in gliomas, Kaplan–Meier analysis was performed to compare the OS between high‐ and low‐risk score group. We observed that the prognosis of patients with high‐risk score was significantly worse than that with low‐risk score (HR=4.30, 95% CI = 3.16–5.85, *p* < 0.0001) (Figure 4A). Furthermore, we also validate the prognostic value of risk score in patients stratified by WHO grade, IDH status, 1p/19q status, and recurrence. As expected, Kaplan–Meier curve showed that patients with high‐risk score had shorter OS than the low‐risk ones in all stratified subgroups, which suggested that risk score was a great predictor to assess the prognosis of patients (Figure S4A–I).

To investigate whether patients with high‐risk scores could benefit from radiotherapy or chemotherapy, we compared the OS of patients with or without radiotherapy and chemotherapy. The Kaplan–Meier curves showed that patients with high‐risk scores were more sensitive to temozolomide treatment and radiotherapy in HGG (Figure 4B–D).

### 3.7 Construction of the nomogram for patients with glioma

To detect the clinicopathological prognosis factors, univariate and multivariate Cox regression analysis were performed. Multivariate analysis showed that there were six independent prognostic risk factors, of which age (*p* = 0.025), grade (*p* < 0.001), recurrence (*p* < 0.001), and risk score (*p* < 0.001) were “risky” factors, while 1p/19q codeletion (*p* = 0.002), chemotherapy (*p* = 0.018) were “protective” factors (Table S4). Then, the risk scores for prognosis were calculated for each patient in 288 patients with glioma. The patients were more trended to poor prognosis when the risk score increased (Figure S5).

**FIGURE 5:**
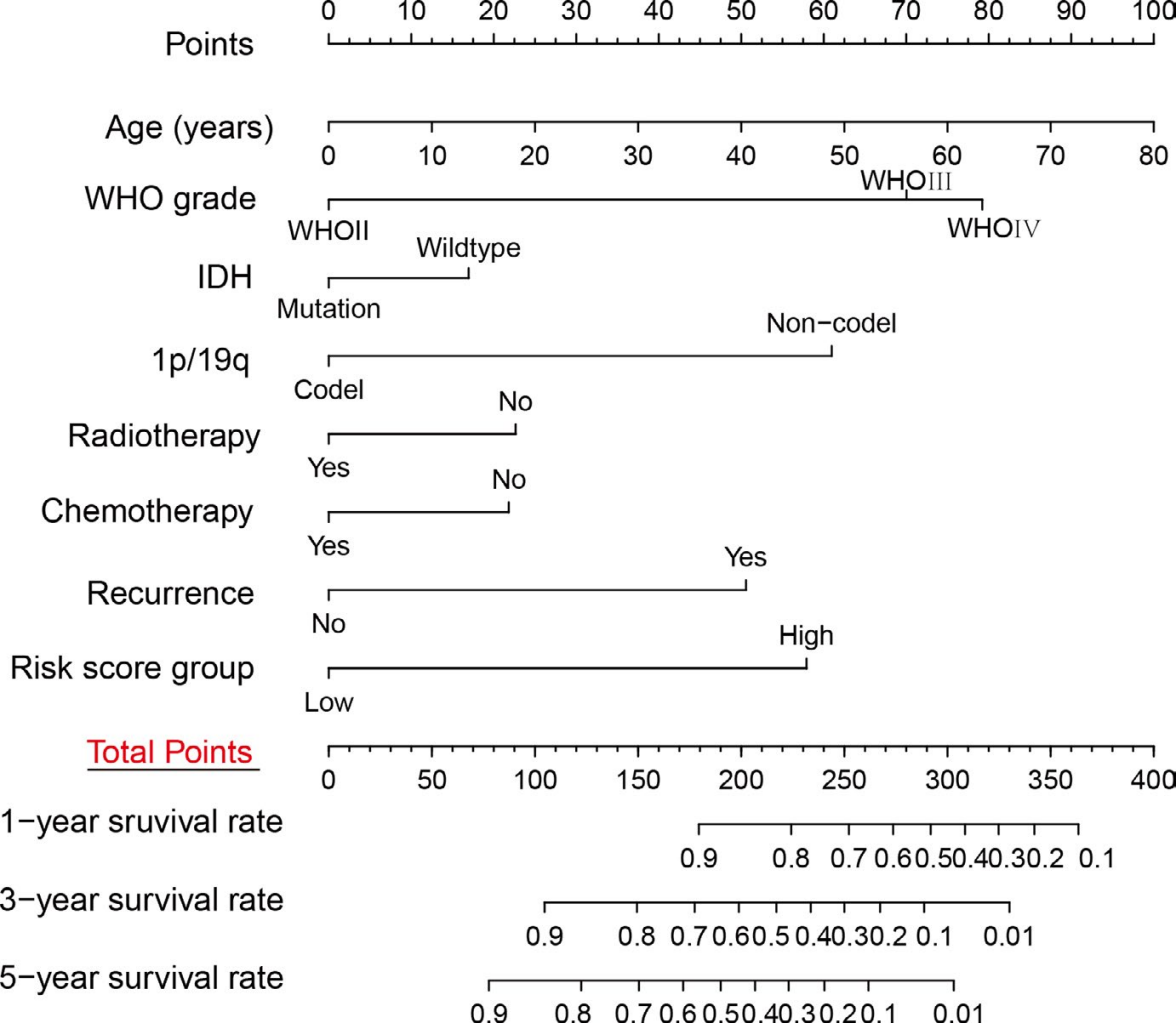
Construction of nomogram for 1‐, 3‐, and 5‐y survival rate in patients with gliomas

Next, a prediction model was constructed by the clinical data and risk score (Figure 5). According to this visual prediction model, we could calculate the total points of each patient, and then obtained the 1‐, 3‐, and 5‐year survival rates. The higher the total points of patient, the worse the prognosis.

### 3.8 Evaluation of nomogram by calibration and time‐dependent ROC curve

**FIGURE 6:**
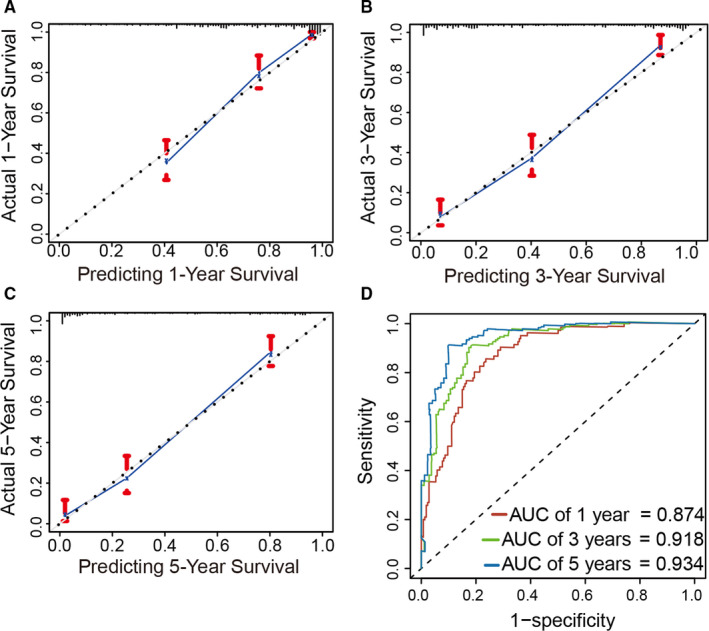
Evaluation of the prediction accuracy of the nomogram. A‐C, Calibration curves showed that the observed and predicted 1‐, 3‐, and 5‐y survival were good agreement. D, Time‐dependent ROC curve for the prediction of the 1‐, 3‐, and 5‐y survival rate based on the nomogram.

To verify the prediction effect of the prognostic model in 288 patients with gliomas, we used the bootstrap self‐sampling method. The *c*‐index is 0.82. Calibration curve and time‐dependent ROC curve were also performed to test the accuracy of the prognostic model. We could see from the calibration curves that the 1‐, 3‐, and 5‐year survival curves predicted by the model were very close to the observed survival curve, which indicated that the nomogram had a high accuracy for prediction (Figure 6A–C). The area under the curve (AUC) of 1‐, 3‐, and 5‐years survival probability were 0.874, 0.918, and 0.934, respectively (Figure 6D).

## 4 DISCUSSION

The treatment of glioma, especially glioblastoma, has always been one of the thorniest problems for clinician. With the development of functional genomic, neuro‐tumor molecules have made great progress recently, and a series of molecular markers which are helpful to the clinical diagnosis and prognosis of gliomas have been found.

In this study, we identified the nine m^6^A‐related genes signature affecting the patients' prognosis by univariate and multivariate Cox regression analysis. We found that the nine m^6^A‐related genes risk score was significantly associated with clinicopathological features. Furthermore, the risk score had a significant correlation with ki‐67 expression, which may be linear. GO and KEGG enrichment analysis identified the nine hub genes signature regulating and controlling RNA splicing, regulation of immune response and vesicle trafficking. These results suggested that the nine genes signature might be involved in the malignant progression of gliomas. Risk score is an independent prognostic factor of patients with glioma and could acted as a new prognostic biomarker. The nine m^6^A‐related genes is expected to become the new targets for further treatment of glioma. We would verify this hypothesis by both *in vitro* and *in vivo* experiments.

The m^6^A methylation is a common internal modification of mRNA in eukaryotes, which is characterized by heredity and reversibility. In 1974, Ronald Desrosiers et al. was the first to report the m^6^A methylation in mRNA.^4^ However, it is not known until now that the mechanism of m^6^A modification has been gradually revealed.^19^ In recent years, increasing studies have confirmed that m^6^A methylation‐related genes, such as ALKBH5, FHL2, DGCR8, YTHDF2, YTHDF3, and PICM8, were overexpressed in tumor tissues and closely related to malignant tumors.^20^ Currently, it has been accepted that this modification is a complex biological process involving multiple m^6^A related genes and mainly affects the tumorigenesis and progression of glioma by mRNA processing, transport, translation, and degradation.^21^ ^,^ ^22^ For instance, Lili Sun et al found a new mechanism of gliomas that overexpressed FHL2 could interact with EGFR and EGFRvIII to increase the stability of their mRNA, which promotes the glioma proliferation.^23^ Previous study showed that YTHDF2 and YTHDF3 included a common YTH domain, which binds to a specific m6A modification site, and then regulates the post‐transcriptional regulation of mRNA by mRNA splicing, translation and localization.^24^ ^,^ ^25^ In addition, Sicong Zhang et al also confirmed that m6A demethylase ALKBH5 is highly expressed in GSCs and promotes the FOXM1 overexpression by increasing the demethylation activity of FOXM1 mRNA, which could promotes the proliferation of GSCs.^10^ The above results suggested that m6A modification is closely related to the phenotype and mechanism of malignant tumors. The results are consistent with the results of our study. However, as we all known, both ALKBH5 and FTO are m6A demethylases. Interestingly, we observed that the upregulation of ALKBH5 acts as a risk factor, while the downregulated of FTO acts as a risk factor in gliomas. Whether ALKBH5 plays a preferential role than FTO in glioma or whether they are specific for modification sites is worth further study.

Moreover, we further construct a clinical prediction model based on the clinicopathological features and m^6^A methylation related genes. Calibration curves and time‐dependent ROC curve were performed to assess the clinical prediction efficiency of nomogram and showed that this prediction model has good prediction efficiency for the 1‐, 3‐, and 5‐year survival rates of patients.

With the implementation of precision medicine, accurate evaluation of patients’ prognosis plays an important role in implementing individualized treatment and improving patients' long‐term prognosis. Although some prognostic biomarkers have been be applied to clinical practice, such as IDH mutation and 1p/19q codeletion, they could not really reflect the individual prognosis.^26^ ^,^ ^27^ Whereas single biomarker lacks ample credibility to predict patients' prognosis, thereby, a model that is composed of more than one biomarker is necessary. We identified nine m^6^A‐related genes as independent prognostic factors in glioma. However, there existed some limitations in this study. The results were lack of the verification of clinical samples or data from other databases. In the future study, the findings will be verified experimentally.

## 5 CONCLUSIONS

In summary, the nine m^6^A‐related genes risk score was identified as a new potential prognostic biomarker in gliomas. The mRNA levels of these genes were highly associated with clinicopathological features of gliomas and might be involved in glioma progression. Additionally, the nomogram based on the nine m6A‐related genes signature and clinicopathological features had good efficacy in predicting the survival probability, which is pivotal to design individual therapy for patients with glioma.

## CONFLICT OF INTEREST

The authors declare no conflict of interest.

## AUTHOR CONTRIBUTIONS

Conception and design: HF‐W, SQ‐Q, and J‐L. Data analysis: ZX‐C, J‐L, and B‐L. Writing and revising: ZX‐C and SQ‐Q. Final approval of manuscript: All authors.

## ETHICS STATEMENT

The study protocol was granted by the Institutional Ethical Review Board of Lishui People's Hospital with a waiver of informed consent.

## Supporting information

Fig S1

Fig S2

Fig S3

Fig S4

Fig S5

Table S1

Table S2

Table S3

Table S4

Supplementary Material

## Data Availability

The data sets analyzed in this study could be obtained from the corresponding author upon reasonable request.
