# Supplementary figures and images for: N6‐methyladenine‐related genes affect biological behavior and the prognosis of glioma
Source: Cancer Med. 2020 Dec 2;10(1):98–108. doi: 10.1002/cam4.3574 (PMC7826482; doi:10.1002/cam4.3574)

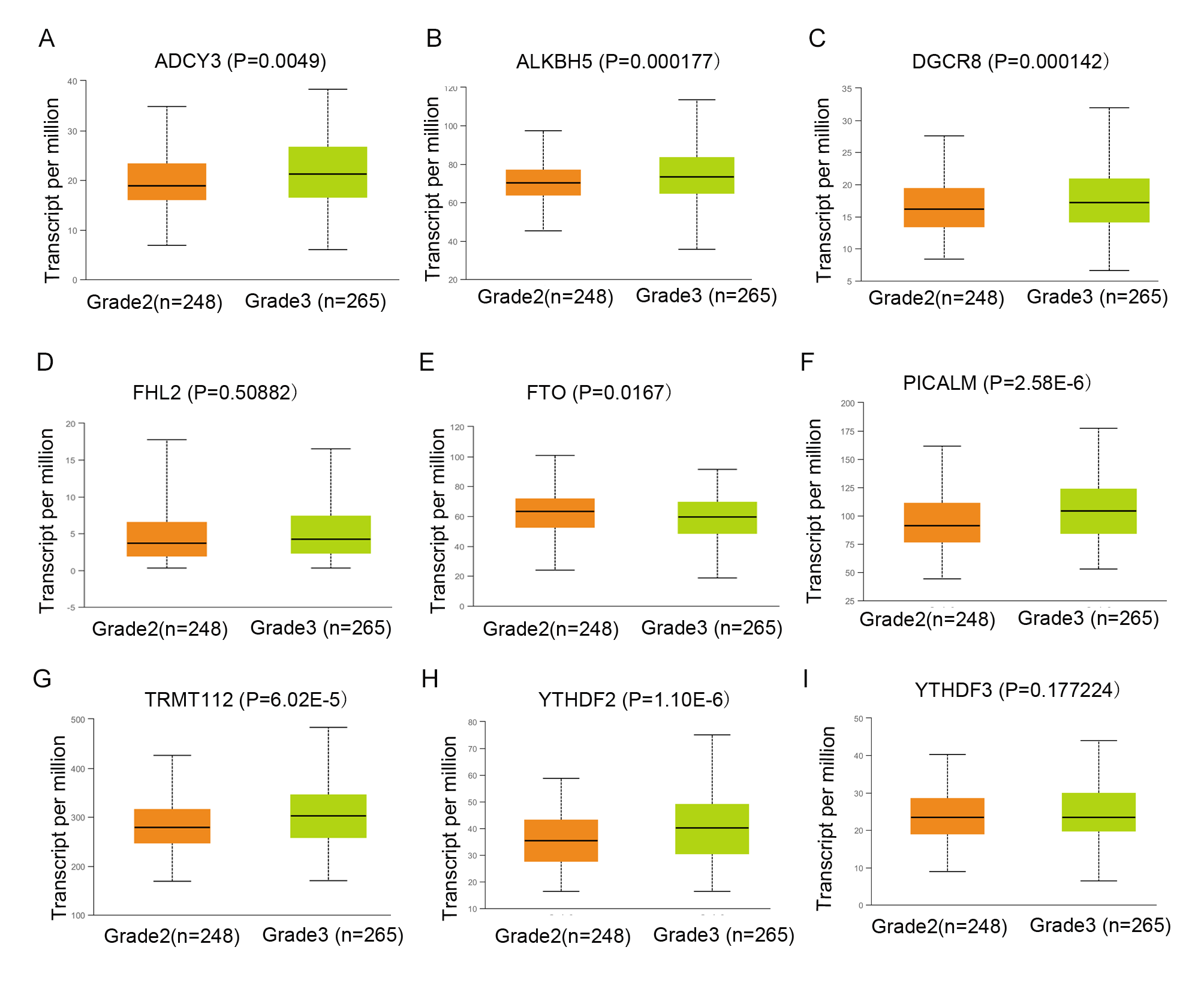

Supplement: Supplementary file 1 — Fig S1 [file CAM4-10-98-s001.tif]

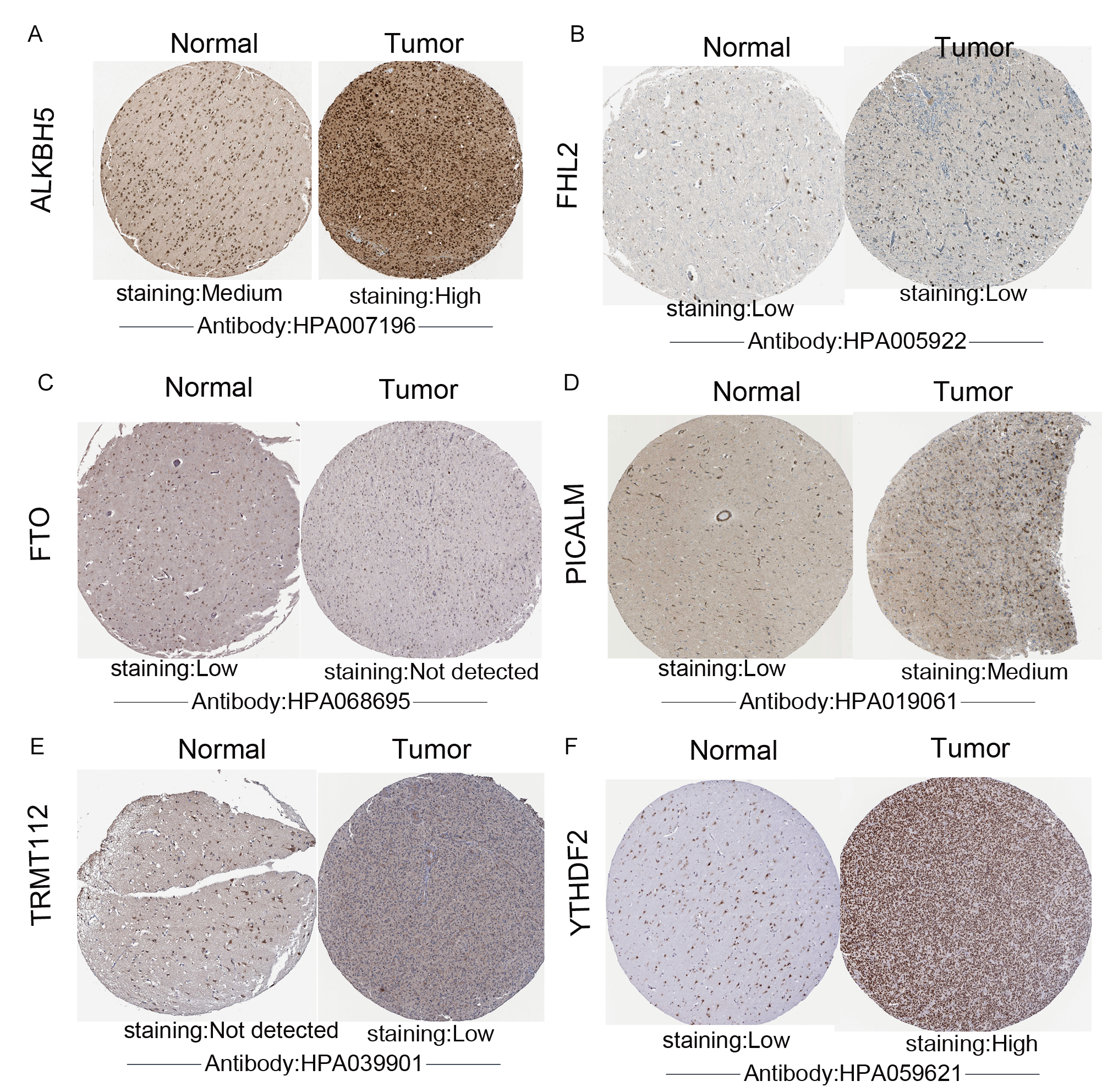

Supplement: Supplementary file 2 — Fig S2 [file CAM4-10-98-s003.tif]

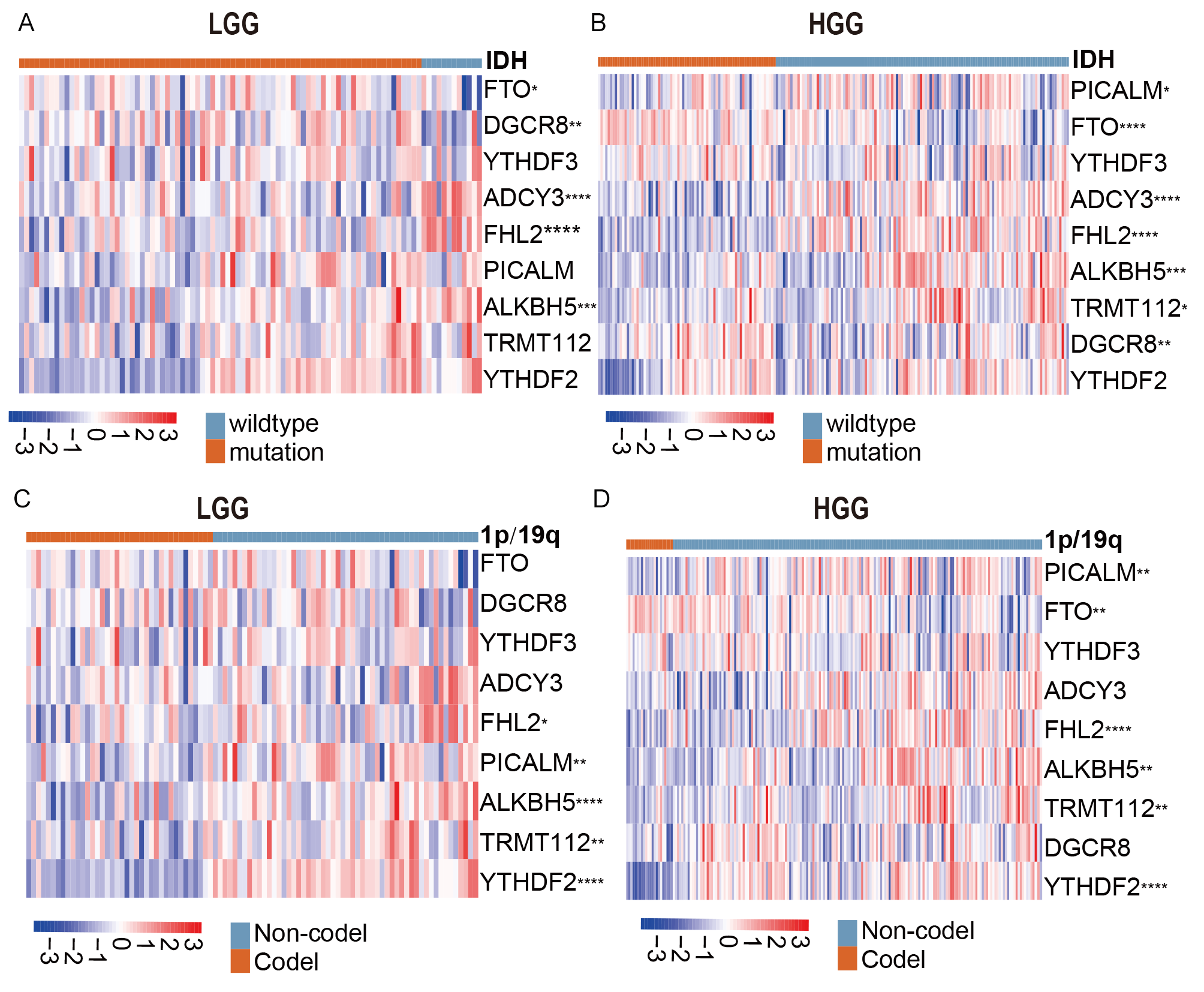

Supplement: Supplementary file 3 — Fig S3 [file CAM4-10-98-s004.tif]

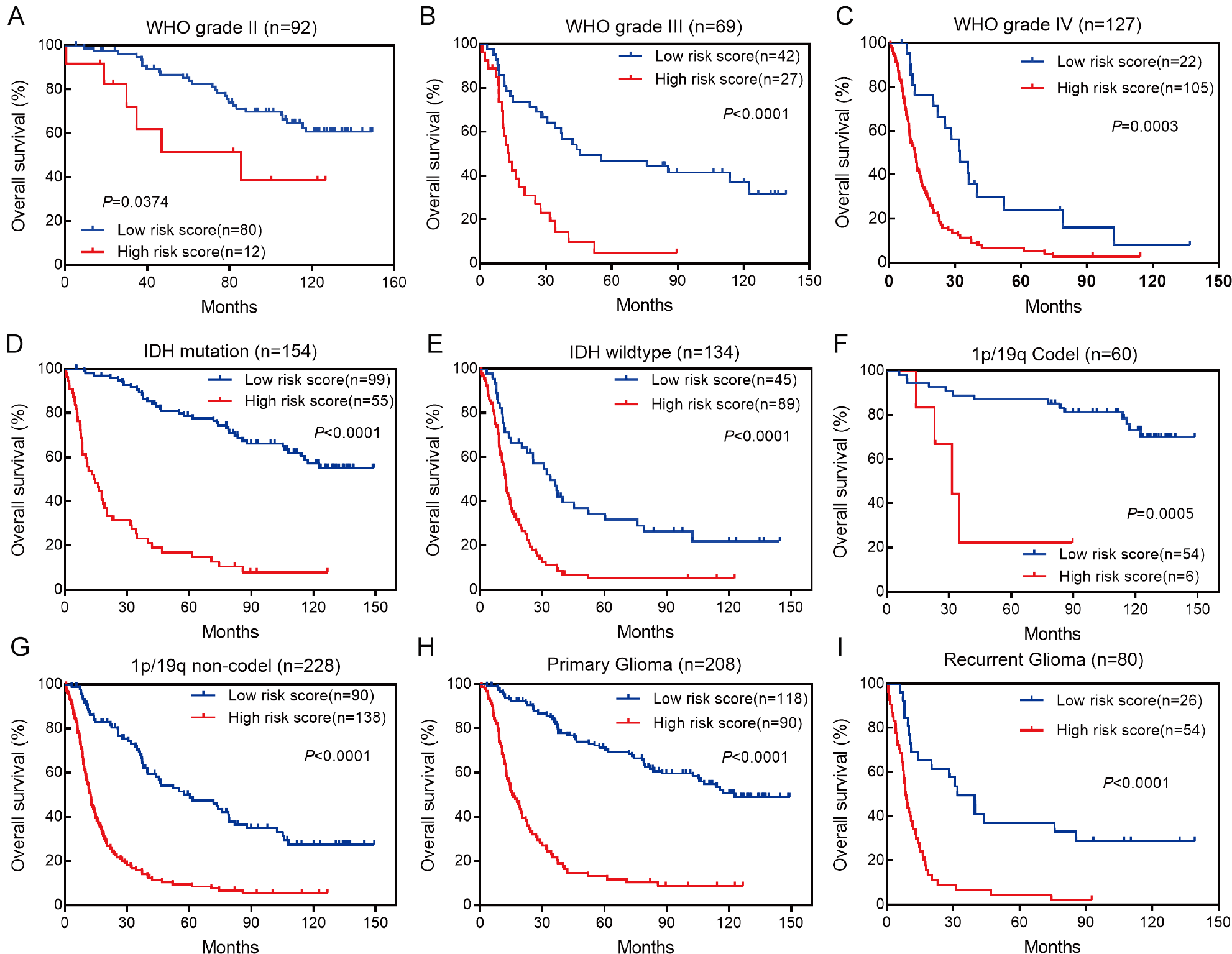

Supplement: Supplementary file 4 — Fig S4 [file CAM4-10-98-s005.tif]

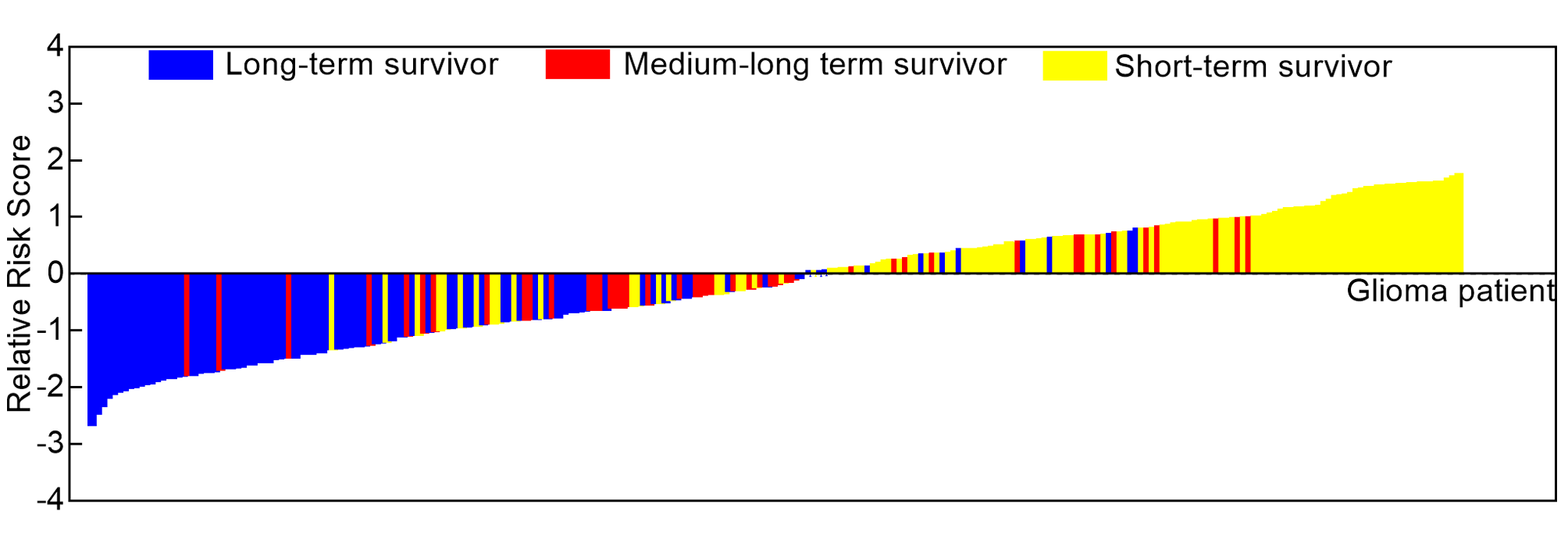

Supplement: Supplementary file 5 — Fig S5 [file CAM4-10-98-s006.tif]
