# Supplementary material for: N6‐methyladenine‐related genes affect biological behavior and the prognosis of glioma
Source: Cancer Med. 2020 Dec 2;10(1):98–108. doi: 10.1002/cam4.3574 (PMC7826482; doi:10.1002/cam4.3574)
Supplement: Supplementary file 6 — Table S1 [file CAM4-10-98-s007.docx]

**TABLE S1** Clinicopathological features of glioma patients.

| **Characteristics** | **Category** | **Number of cases** | **(%)** |
| --- | --- | --- | --- |
| **Age(years)** | <20 | 8 | 2.8 |
|  | 20-40 | 107 | 37.2 |
|  | 40-60 | 141 | 49 |
|  | ≥60 | 32 | 11.1 |
| **Gender** | Male | 180 | 62.5 |
|  | Female | 108 | 37.5 |
| **WHO grade** | WHO II | 92 | 31.9 |
|  | WHO III | 69 | 24 |
|  | WHO IV | 127 | 44.1 |
| **IDH** | Mutation | 154 | 53.5 |
|  | Wildtype | 134 | 46.5 |
| **1p/19q** | Codel | 60 | 20.8 |
|  | Non-codel | 228 | 79.2 |
| **Radiotherapy** | Yes | 244 | 84.7 |
|  | No | 44 | 15.3 |
| **Chemotherapy** | Yes | 171 | 59.4 |
|  | No | 117 | 40.6 |
| **Recurrence** | Yes | 208 | 72.2 |
|  | No | 80 | 27.8 |
| **Overall survival**  **(months)** | <12 | 88 | 30.6 |
|  | 12-36 | 77 | 26.7 |
|  | 36-60 | 28 | 9.7 |
|  | ≥60 | 95 | 33 |
