# Supplementary material for: N6‐methyladenine‐related genes affect biological behavior and the prognosis of glioma
Source: Cancer Med. 2020 Dec 2;10(1):98–108. doi: 10.1002/cam4.3574 (PMC7826482; doi:10.1002/cam4.3574)
Supplement: Supplementary file 7 — Table S2 [file CAM4-10-98-s008.docx]

**TABLE S2** Univariate and multivariate Cox-regression analysis of m^6^A-related genes affecting the prognosis of patients.

| **Gene** | **Univariate Cox analysis** | | | **Multivariate Cox analysis** | | |
| --- | --- | --- | --- | --- | --- | --- |
|  | **HR** | **95%CI** | ***P*-value** | **HR** | **95%CI** | ***P*-value** |
| ADCY3 | 5.45 | 2.54-11.7 | <0.001 | 0.22 | 0.06-0.79 | 0.019 |
| ALKBH5 | 54.12 | 22.26-131.61 | <0.001 | 28.22 | 6.37-125.04 | <0.001 |
| DGCR8 | 13.6 | 6.02-30.72 | <0.001 | 5.01 | 1.09-23.06 | 0.039 |
| FHL2 | 6.92 | 2.42-19.77 | <0.001 | 0.11 | 0.02-0.57 | 0.008 |
| FTO | 0.03 | 0.01-0.07 | <0.001 | 0.12 | 0.02-0.75 | 0.023 |
| PICALM | 8.56 | 3.56-20.59 | <0.001 | 7.72 | 1.78-33.51 | 0.006 |
| TRMT112 | 32.98 | 14.01-77.6 | <0.001 | 174.46 | 7.14-4265.7 | 0.002 |
| YTHDF2 | 29.18 | 15.78-53.97 | <0.001 | 5.8 | 1.18-28.47 | 0.030 |
| YTHDF3 | 5.97 | 2.06-17.33 | 0.001 | 14.93 | 1.79-124.48 | 0.012 |

HR, hazard ratio; CI, confidence interval.
