# Supplementary material for: N6‐methyladenine‐related genes affect biological behavior and the prognosis of glioma
Source: Cancer Med. 2020 Dec 2;10(1):98–108. doi: 10.1002/cam4.3574 (PMC7826482; doi:10.1002/cam4.3574)
Supplement: Supplementary file 8 — Table S3 [file CAM4-10-98-s009.docx]

**TABLE S3** The biological function and hazard regression coefficients of the 9 m^6^A-gene signature.

| **Signature** | **Description** | **Category** | **Coefficient** | **Relevance score** |
| --- | --- | --- | --- | --- |
| FTO | FTO Alpha-Ketoglutarate Dependent Dioxygenase | Protein Coding | -2.12 | 22.68 |
| YTHDF2 | Vir Like M6A Methyltransferase Associated | Protein Coding | 1.76 | 16.17 |
| YTHDF3 | YTH N6-Methyladenosine RNA Binding Protein 3 | Protein Coding | 2.70 | 8.73 |
| FHL2 | Four And A Half LIM Domains 2 | Protein Coding | -2.21 | 8.4 |
| PICALM | Phosphatidylinositol Binding Clathrin Assembly Protein | Protein Coding | 2.04 | 8.4 |
| ALKBH5 | AlkB Homolog 5, RNA Demethylase | Protein Coding | 3.34 | 8.21 |
| DGCR8 | DGCR8 Microprocessor Complex Subunit | Protein Coding | 1.61 | 2.82 |
| ADCY3 | Adenylate Cyclase 3 | Protein Coding | -1.51 | 2.45 |
| TRMT112 | TRNA Methyltransferase Subunit 11-2 | Protein Coding | 5.16 | 1.36 |
