# Supplementary material for: N6‐methyladenine‐related genes affect biological behavior and the prognosis of glioma
Source: Cancer Med. 2020 Dec 2;10(1):98–108. doi: 10.1002/cam4.3574 (PMC7826482; doi:10.1002/cam4.3574)
Supplement: Supplementary file 9 — Table S4 [file CAM4-10-98-s010.docx]

**TABLE S4** Univariate and multivariate Cox-regression analysis of clinicopathological features affecting the prognosis of patients.

| **Parameters** | **Univariate Cox analysis** | | | **Multivariate Cox analysis** | | |
| --- | --- | --- | --- | --- | --- | --- |
|  | **HR** | **95%CI** | ***P*-value** | **HR** | **95%CI** | ***P*-value** |
| **Age** | 1.03 | 1.02-1.05 | <0.001 | 1.02 | 1.00-1.03 | 0.025 |
| **Gender** | 1.03 | 0.77-1.38 | 0.828 |  |  |  |
| **Grade** | 2.84 | 2.35-3.45 | <0.001 | 1.65 | 1.28-2.11 | <0.001 |
| **IDH** | 0.35 | 0.26-0.47 | <0.001 | 0.69 | 0.48-1.00 | 0.053 |
| **1p/19q** | 0.18 | 0.11-0.29 | <0.001 | 0.43 | 0.25-0.74 | 0.002 |
| **Radiotherapy** | 0.52 | 0.36-0.75 | <0.001 | 0.76 | 0.51-1.12 | 0.165 |
| **Chemotherapy** | 1.75 | 1.30-2.37 | <0.001 | 0.67 | 0.48-0.93 | 0.018 |
| **Recurrence** | 2.63 | 1.95-3.55 | <0.001 | 2.45 | 1.78-3.49 | <0.001 |
| **m6A risk score** | 5.27 | 3.85-7.21 | <0.001 | 1.57 | 1.36-1.82 | <0.001 |

HR, hazard ratio; CI, confidence interval.
