# Supplementary material for: N6‐methyladenine‐related genes affect biological behavior and the prognosis of glioma
Source: Cancer Med. 2020 Dec 2;10(1):98–108. doi: 10.1002/cam4.3574 (PMC7826482; doi:10.1002/cam4.3574)
Supplement: Supplementary file 10 — Supplementary Material [file CAM4-10-98-s002.docx]

**Figure S1** The expression of m^6^A-related genes in different glioma. (A-I) TCGA data analysis of ADCY3, ALKBH5, DGCR8, FHL2, FTO, PICALM, TRMT112, YTHDF2, and YTHDF3 between WHO grade 2 and grade 3 glioma.

**Figure S2** Comparison of the differential expression encoded by the significantly m^6^A-related genes between normal tissue and glioma tissue in Human Protein Atlas database. (A-F) The typical IHC plots of ALKBH5, FHL2, FTO, PICALM, TRMT112, and YTHDF2 in normal and glioma tissue. There were no related IHC samples of DGCR8, YTHDF3, and ADCY3 in this database.

**Figure S3** The mRNA expression of m^6^A-related genes and their correlation with IDH and 1p/19q status in LGG and HGG. (A-B) The expression levels of nine-m6A-related genes in LGG and HGG with different IDH status; (C-D) the expression levels of nine-m6A-related genes in LGG and HGG with different 1p/19q status.

**Figure S4** Comparison of overall survival between glioma patients with high- and low-risk scores. (A-C) Kaplan–Meier survival curves with different WHO grade groups; (D-E) Kaplan–Meier survival curves with IDH-mutation and IDH-wild-type groups; (F-G) Kaplan–Meier survival curves with 1p/19q codel and non-codel groups; (H-I) Kaplan–Meier survival curves with primary and recurrent glioma groups.

**Figure S5** Waterfall plots of risk score and prognosis of individual patient (Short-term survivor<24 months, 24≤ Medium-long-term survivor<60 months, Long-term survivor ≥60 months).
